# Editorial: Biomechanics, aging, exercise and other interventions

**DOI:** 10.3389/fbioe.2022.1056547

**Published:** 2022-11-07

**Authors:** Rafael Reimann Baptista, Marcus Fraga Vieira, Rezaul Begg, Chiarella Sforza

**Affiliations:** ^1^ Pontifical Catholic University of Rio Grande do Sul, Porto Alegre, Brazil; ^2^ Universidade Federal de Goiás, Goiânia, Brazil; ^3^ Victoria University, Melbourne, VIC, Australia; ^4^ Department of Biomedical Sciences for Health, Università degli Studi di Milano, Milan, Italy

**Keywords:** aging, older adults, exercise, interventions, kinesiology

Physiological and biomechanical changes observed during human aging are related to several factors, but alterations in physical health and deficits in motor control are among the most frequent. In this Research Topic we collected some investigations describing the normal modifications occurring in older adults, as well as the effect of specific interventions to counteract the side effects and complications of aging.

Due to the increase in life expectancy, people over the age of 65 years are increasing rapidly. According to a 2019 worldwide estimate, about 16% of the population will be older adults by 2050 compared to 9% less than 5 years ago. In the same timespan (2019–2050) the number of people aged 80 years or over is expected to increase from 143 million to 426 million (United Nations, 2022).

Non-pharmacological treatments, such as exercise, have been highlighted in several studies. Exercise can be proposed in multiple forms, from sport, outdoor walking, indoor gyms, and dedicated classes to private home facilities, but, on all occasions, there should be control of the performance and the effect of the intervention. Before the COVID-19 pandemic, controlled training and rehabilitation coaching were directly provided during the classes or in dedicated laboratories, using conventional optoelectronic motion-capture systems or inertial measurement units. Lockdown weeks showed the necessity to continue to control the effects of home physical exercise on the locomotor system using new, simplified, and low-cost devices. The effectiveness of such devices was evaluated in the current Research Topic. For example, Lee and Park devised a 2-TV camera system that can effectively be used for assisting home exercises.

Delbes et al. proposed a head-mounted display for overground walking during fully immersive virtual training sessions in older adults. The authors showed that older adults adopted the same gait adaptability behavior despite the use of a real vs. virtual environment. Besides, the older adults that participated in the study exhibited good acceptance of the virtual reality device, opening the possibility to design training programs in virtual reality to prevent falls in older adults.

Of course, motion capture is just one of the many tools that can help exercise planning and evaluation. The effectiveness of dynamometers, force platforms, surface EMG, and body composition instruments was investigated in the current Research Topic (Čretnik et al.). Also, exercise planning in older adults should consider age-related characteristics and cannot be directly imported from protocols devised for younger persons.

For example, the most classical form of exercise is sport, and indeed, sports practice should be safe at all ages, with physiological modifications contributing to athletic performance. Unfortunately, some sports include activities with potentially negative implications that can jeopardize an athletic career and should be known by athletes, coaches, and health professionals for screening and injury prevention perspectives. Muscles, tendons, cartilages, and bones are frequently involved, as reported for Taekwondo (Xu et al.) and the alpine sky (Götschi et al.).

During running, fatigue can affect joint biomechanics dramatically. Quan et al. found that there was no proximal shift in knee joint mechanics in amateur female runners after prolonged running. The variations in running fatigue were related to the redistribution of joint work. The authors suggest that to reduce the risk of injuries associated with long-distance running, athletes should also train their muscles.

Tennis training is another popular sport used for fitness maintenance during the lifespan. While evaluating children between the ages of 7 and 8 beginning to play tennis, Wang et al. observed that when introduced to childhood tennis beginners during a sensitive period, regular tennis training combined with a neuromuscular training program could produce greater improvement in a player’s sprint and ability to change direction than tennis class intervention alone.

Fatigue is not the only aspect that a physiologist should consider when studying human performance. The Recovery Rate is also important, and this was addressed by Markus et al.. They found that following an exercise-induced muscle damage downhill protocol, young and middle-aged male amateur athletes recover comparably. These findings suggest that trainees of both ages can use similar recovery strategies after completing an aerobic-based exercise-induced muscle damage protocol.

For assessing knee joint disorders, not only deficits in quadriceps flexibility are a risk factor, but their stiffness should also be considered. Chang et al. have shown that patients with knee osteoarthritis (KO) exhibited greater vastus lateralis (VL) stiffness compared to age-matched healthy adults. Furthermore, the authors found a positive correlation between VL stiffness and the Western Ontario and McMaster University’s Osteoarthritis Index in patients with KO. These findings suggest an additional evaluation of quadriceps muscle for a proper KO patient follow-up.

In another study published in the current Research Topic, Chen et al. analyzed the effect of plantar-flexion motion on the stiffness of the lumbar and lower limb tissues. Healthy humans participated in this study and performed isometric plantar flexion against different resistance conditions. Their data showed a significant effect of isometric plantar flexion on the stiffness of the lumbar soft tissue and gastrocnemius.

Our Research Topic was not sorely dedicated to exercise physiology. Clinical biomechanics was also addressed as can be observed by the study of De La Fuente et al.. They present interesting data suggesting that crutches on the elbows change how the gluteus medius (GM) activates when climbing stairs. The number of crutches, the lateral usage of the crutches, and whether the leg is loaded or unloaded when going up the stairs were analyzed in detail. Their findings may help those who currently use or plan to use crutches to activate their GM.

Biomechanical modeling was also addressed in this Research Topic. Kumar et al. studied the sit-to-stand adaptations due to muscle strength deficits and the corresponding assistance trajectories adopted in those adaptations using an open-loop single shooting optimization framework and musculoskeletal models. The authors showed that vasti muscle saturation leads to reduced activation of hamstring muscles and gluteus maximus saturation, suggesting that vasti muscle weakness is responsible for sit-to-stand failure. Besides, external assistance can be used when needed to complement strength deficits for a successful sit-to-stand. Such a model can help to design an intervention and novel sit-to-stance assistance devices.

Advanced modeling techniques such as machine learning are being applied to predict internal biomechanical data and as well as data patterns. Using the deep learning method, Boukhennoufa et al. have predicted internal knee abduction impulses from body kinematics and kinetics data. The study found transfer learning to be the best-performing model, achieving a mean absolute percentage error of 8.28%. Since knee joint abduction moments provide an indirect measure of knee joint loading during locomotion, this type of machine learning-based prediction offers the possibility of many clinical applications.

Other authors have proposed challenging situations to improve postural control in older adults. Tsai et al. have studied the effects of stroboscopic vision (SV) on postural fluctuations and cortical processing during stance. SV induced greater postural fluctuations and reduced EEG power in the mid-frontal theta cluster but enhanced in the visual dorsal and frontal-occipital loops of the right hemisphere. The authors concluded that SV adds challenge to postural control and suggest that older adults shift their great dependence on visual inputs to control with more non-visual awareness.

Regarding the improvement of older adults’ fitness, strength training is considered as one of the best interventions for this population. Čretnik et al. performed a Systematic Review with Meta-Analysis to examine how traditional or concentric exercise affects older persons’ muscle strength, body composition, and functional performance in comparison to eccentric exercise modalities. Their research showed eccentric exercise is preferable to concentric exercise, or at least just as effective, for maintaining general health and function in older persons.

In this sense, Zhao et al. have studied the effects of low-intensity resistance exercise combined with blood flow restriction in hypertension older adults. They showed that compared to high-intensity resistance exercise and low-intensity resistance exercise, low-intensity resistance exercise combined with blood flow restriction could effectively decrease systolic pressure in hypertension older adults performing quadriceps femoris resistance exercise after 12 weeks of training.

Surgical treatment sometimes is a necessary intervention, especially in older adults. Total hip arthroplasty, dubbed “the operation of the century,” has been proven to be incredibly successful for decades. But today’s patients want more from their surgeons; if not adequately advised, they now anticipate a painless joint restoration and a quick return to work and sports. Bender et al. discovered evidence to support the need to give patients critical feedback regarding their expectations for returning to work and athletic activities to address this topic. Patients returning to more demanding sports should be closely watched and counseled to prevent overloading as much as possible.

The importance of an active life as well as the relevant role of physical activity in the health and well-being of people of all ages is now well recognized by the scientific, medical, and civil society in general. Physical activity plays an important role in healthy aging, prolonging life by reducing the onset of chronic diseases that affect both physical and mental health.

Overall, the investigations collected by the current Research Topic provided a set of new information that may help the definition of novel exercise protocols (Čretnik et al.; Zhao et al.) specifically suited for elderly people such as the successful use of virtual reality to improve postural control (Delbes et al.) and of stroboscopic vision (Tsai et al.). Activity can then be detected by low-cost devices thus helping to test and training (Lee and Park). The Research Topic also introduced several biomechanical models of the knee joint and the lower limb muscles that represent the theoretical background for the definition of safe and effective training in both healthy and diseased people (Wang et al.; Markus et al.; Chang et al.; Kumar et al.; De La Fuente et al.). From this point of view, current investigations are more and more studying the active part of the locomotor system also thanks to suitable instruments for their assessment (Chen et al.; De La Fuente et al.).

Another important result is the growing attention to injury prevention and evaluation (Xu et al.; Götschi et al.), with a special focus on the effects of fatigue (Quan et al.; Bender et al.). One of the most advanced study mixed biomechanics and artificial intelligence using a deep learning method to predict internal knee forces (Boukhennoufa et al.). The method can be potentially applied to several contexts providing more reliable and predictive models of the joints and muscles. It represents the first step for future investigations.
